# Management of Practice

**Published:** 1892-06

**Authors:** W. H. Whitslar


### Management of Practice.

BY DR. W. H. WHITSLAR.

(Read before Cleveland Dental Society, May 2. 1892.)

The practice of dental surgery means—the treatment of diseases of the dental tissues and adjacent parts, also, includes the making and adjusting of artificial substitutes when such parts are lost.

This means much. Our professional duties have become so enlarged within the last decade, by various improvements that have been learned, that the extent of dental practice seems almost unlimited.

Duty commands us to work, and labor has solved many niceties of practice that render mankind ever thankful for the age in which he lives, and yet, principles of thousands of years ago remain the same as ever requiring only the genius of the nineteenth century to evolve.

Americans may well pride themselves upon the fact of their leading the world in advancement of dental science. Necessity of this advancement was recognized, and too, environments have assisted in this wealth of progress. This led our European friends to a charming race, and in full view of the fact that they lead precedence by reason of age and many trials, our spirited American wears the crown of victory. In manifestation of this, students crowd our dental colleges from every quarter of the globe, giving intimation that the seat of learning is in American schools. Compatible with this invasion of students, our investigators and inventors are unceasingly increasing our knowledge because of the obligations resting upon them.

The statement that the dentist operates upon living tissues of human beings, is *prima facie* evidence of a stupendous obligation. Many times the life of a patient is weighed in the balance, and a mite of ignorance may sever the life-line. The laws of the community should be indicative of a Nation's regard for the lives of its subjects, and finally, the people themselves should figure for a happy and lengthy stay upon earth, rather than one of torture from poor service and horrible treatment by the hands of licentious quacks.

The first natural emolument then necessary to a successful management of a dental practice is *education.* Many dentists seem to have a fine practice; their offices are crowded with the elite, they live and flourish well, their personal wants are gratified in every way; they have plenty of money. This shows a good business management which may be that and that only. Or, if combined with this, recognized ability may take equal or more than equal share; how often are we led to ask "does Dr. Blank give competent service for value received?"

Some good operators are ruined by having too many patients. Overcrowding naturally leads to hasty operations with neglect of important and necessary conditions to be fulfilled, and ere long, complaints are made that Dr. Blank is not successful in the art of saving teeth. It is far better to have a limited number of patients and successfully manage these, than to spread ruination among many.

The value of teeth is inestimable, and their preservation by competent masters of this art, a grand science. Who would not be a master of what he professes? Yet there are shysters in every hamlet denuding his good name of every vestige of manliness, by proposing to curtail the expenses of those that seek his service. Conscience is not a part of his education, and a credulous populace are deceived, ruined, and the educated dentist suffers by an unjust comparison. So I must argue that to have successful management one must have education, which should begin as early in life as when duties for right and against wrong are conceived. You may then lead the individual through the various schools of secular and moral learning, and his mind may be enhanced by collegiate courses in literary and scientific schools. Love of art and music may be put into practical use in training for the practice of dentistry. Manual training is of paramount importance as a supplement to an educated mind. In fact there is such a correlation of accessions to one's education, that it is plainly evident that a broad understanding will give one prestige over the the contracted mind of the uneducated dentist.

It is true that we often see a good dentist who has not had school advantages, experience has been his teacher, purchased with the price of many years. Energy has been his main support. The same energy, in use with brains to direct affairs, will always produce the best dentist. There are other items in the conduct of practice to be considered. Let us imagine our student graduated from a reputable college. He locates in a desirable community where he expects to live and become a good citizen. His office is his first consideration. It must be centrally located and of easy access. His rooms should be commodious and convenient for his work; good windows are among the first considerations. These may face any direction, except the direct west. Economy may be found in good carpets and furniture. Elegant furniture should have a place in your home rather than the office, and so should your high priced paintings be placed for your wife and family to enjoy; the office is for business and not for a parlor. Parlors are nowadays used by quacks as offices to make up in appearances what they lack in virtue. The office should be pleasant of course, but it is better to give it the appearance of a professional one. This may be done without an exhibition of skulls and teeth, or specimens that will excite morbid curiosity. Instruments and appliances best suited for your purpose are the greatest consideration in opening an office. Spend more money for these than you do in fixing up your office is wholesome advice. For a dental operating chair and other necessities that will last many years, it is cheaper to get the best at the very start, and leave the minor appurtenances come in due time, as they certainly must with less trouble. There are many other things that might be said in regard to office equipment. When ready for business hang out your sign; don't plaster the walls with your placard. A sign of simple construction in a desirable place is sufficient. Patients will hear of you and now your work begins.

The practice of what has been learned at college requires prudence, also much moral courage. Care of your eyes and physical welfare is part of the stock you carry. Drunkenness will not be tolerated. Tobacco is an abomination. These and a thousand other things are required of us, and the most of them are not hard to carry out unless already in the toils of vices. Cheerfulness should be your demeanor when you invite the patient to be seated in your operating chair. Avoid coarse stories or any indication of not being a gentleman, but some pleasantry may serve to make the patient feel at ease. Any frivolity will lessen your chances of scientific success. Intimacy breeds contempt. Observe the private rights of the patient thoroughly. It is perfectly proper to let the patient know or see you wash your hands before touching his mouth, and it is a satisfaction to have a clean napkin, or towel, placed over the chest and shoulders, also one upon the head rest. These are slight observations of the management preparatory to an operation. Upon the capability, skill or artifice of the operator to detract from painful operations, his success is often dependent. No two persons are alike, and it may be that with one patient he may not succeed well, whilst in the hands of another dentist, the operaticns prove successful. Happy expedients are necessary very often to master control of the patient. My method, quite often, when a patient insists she is very nervous, is to humor her out of that belief. Each patient is a study, and how to manage the various idiosyncrasies, is indeed quite a large part of the control of practice. Out of the office the dentist should observe proper demeanor, such as become a professional man.

Whatever is worth doing at all is worth doing well, and so we believe that deviations, which are far from our calling, such as politics and any partisan movements, will not aid practice. Studies correlated to the science of dentistry are great aids as well as pastimes.

In conclusion, (leaving much to be said by those who will discuss this subject), there remains one item that dentists invairably almost neglect, and that is collection of fees. Dentists are poor collectors. We often hear it said that it is easier to do the work than collect the fee. Upon this part of practice I am weak myself, and don't practice what I preach; but it is certainly correct to send statements of accounts immediately after service is rendered. If dentists everywhere would practice this, we would revolutionize the ideas of your patients, and greater respect for our calling would accrue in the end.

